# SNP@lincTFBS: An Integrated Database of Polymorphisms in Human LincRNA Transcription Factor Binding Sites

**DOI:** 10.1371/journal.pone.0103851

**Published:** 2014-07-30

**Authors:** Shangwei Ning, Zuxianglan Zhao, Jingrun Ye, Peng Wang, Hui Zhi, Ronghong Li, Tingting Wang, Jianjian Wang, Lihua Wang, Xia Li

**Affiliations:** 1 College of Bioinformatics Science and Technology, Harbin Medical University, Harbin, China; 2 The Second Affiliated Hospital, Harbin Medical University, Harbin, China; South Texas Veterans Health Care System and University of Texas Health Science Center at San Antonio, United States of America

## Abstract

Large intergenic non-coding RNAs (lincRNAs) are a new class of functional transcripts, and aberrant expression of lincRNAs was associated with several human diseases. The genetic variants in lincRNA transcription factor binding sites (TFBSs) can change lincRNA expression, thereby affecting the susceptibility to human diseases. To identify and annotate these functional candidates, we have developed a database SNP@lincTFBS, which is devoted to the exploration and annotation of single nucleotide polymorphisms (SNPs) in potential TFBSs of human lincRNAs. We identified 6,665 SNPs in 6,614 conserved TFBSs of 2,423 human lincRNAs. In addition, with ChIPSeq dataset, we identified 139,576 SNPs in 304,517 transcription factor peaks of 4,813 lincRNAs. We also performed comprehensive annotation for these SNPs using 1000 Genomes Project datasets across 11 populations. Moreover, one of the distinctive features of SNP@lincTFBS is the collection of disease-associated SNPs in the lincRNA TFBSs and SNPs in the TFBSs of disease-associated lincRNAs. The web interface enables both flexible data searches and downloads. Quick search can be query of lincRNA name, SNP identifier, or transcription factor name. SNP@lincTFBS provides significant advances in identification of disease-associated lincRNA variants and improved convenience to interpret the discrepant expression of lincRNAs. The SNP@lincTFBS database is available at http://bioinfo.hrbmu.edu.cn/SNP_lincTFBS.

## Introduction

Large intergenic non-coding RNAs (lincRNAs) are recently emerging as a novel class of functional non-coding RNAs, which are more than 200 nucleotides in length, derive from the intervals between protein-coding genes, have similar exon-intro-exon structure, but lack of protein-coding capacity [1]. As yet, the quantity of discriminated human lincRNA transcripts continue to increase [2], and many of them have been found to play important roles in multiple biological processes, including epigenetic regulation of protein-coding gene expression [3]–[5] and crucial action in development process [6]. Emerging evidence has also demonstrated that numerous lincRNAs were associated with a wide range of human diseases [7].

Recently, several profiling studies have revealed that dysregulated expression of lincRNAs was involved in several forms of human cancer [8]. For example, a study has reported that the expression level of lincRNA *PCGEM1* was higher in prostate tumor specimens than in matched normal tissues [9]. LincRNA *HOTAIR* (HOX antisense intergenic RNA) can be regard as an independent cancer prognostic marker due to its significantly overexpression in breast cancer, hepatocellular cancer, colorectal cancer and laryngeal squamous cell carcinoma [10]–[12]. Another highly abundant lincRNA *MALAT1* (also known as *NEAT2*) is originally identified as a marker for lung cancer metastasis; its expression is strongly regulated in many tumor entities including lung adenocarcinoma and hepatocellular carcinoma [13], [14]. In addition, it has been demonstrated that up-regulation of a lincRNA *HULC* is highly associated with the incidence of hepatitis B virus (HBV) infection [15]. However, despite a number of lincRNAs having aberrant expression in disease states, the causality that affects the expression abundance of lincRNAs has yet to be completely understood.

Previous studies have shown that single nucleotide polymorphisms (SNPs) in transcription factor binding sites (TFBSs) of protein-coding genes could affect gene expression by altering transcription factor binding, and participated in human diseases [16]–[20]. A recent study on a tumor suppressor lincRNA has also demonstrated that a SNP (rs944289) could predispose to papillary thyroid carcinoma through dysregulating lincRNA (PTCSC3) expression by decreasing the binding activity of both C/EBPα and C/EBPβ [21]. Thus, SNPs in the human lincRNA TFBSs can act as a set of functional variants, which may disrupt transcription factor binding, resulting in the diversity of lincRNA expression and, potentially, diverse diseases.

Furthermore, with the advent of high-throughput technologies, large-scale lincRNA annotation data, SNP data, predicted and experimentally supported TFBSs data have been generated. This provides a great opportunity to systematically identify SNPs in the human lincRNA TFBSs. For example, in the new update of NONCODE database, the lincRNA data set were expanded by collection of newly identified lincRNAs from published literatures and integration of the latest version of RefSeq and Ensembl [22]. LncRNADisease database collected experimentally supported lncRNA-disease associations and lncRNA interacting partners at various molecular levels [23]. ChIPBase database was developed to annotate and identify TFBSs and transcriptional regulatory relationships of lncRNAs and miRNAs from ChIP-Seq data [24]. In addition, the ENCODE project has compiled a large number of ChIP-Seq experiments for many human TFs in different cell lines and tissues [25]. Enriched peak regions of these ChIP-Seq data can be mapped to the promoter regions of lincRNAs, which facilitate the discovery of experimentally supported TFBSs of human lincRNAs in different cell lines and tissues, and also give us a better opportunity to identify SNPs in lincRNA TFBSs for a cell line of interest.

Therefore, to provide a beneficial annotation of these potential functional variants in human TFBSs, we developed a SNP@lincTFBS database for integrating and annotating functional SNPs in predicted lincRNA TFBSs. We identified 6,665 SNPs occurring in 6,614 TFBSs of 2,423 human lincRNAs, and provided a comprehensive and useful resource of candidate SNPs relevant to the aberrant expression of lincRNAs. The SNP@lincTFBS database will be helpful to identify functional SNPs of lincRNAs in the level of transcription and contribute to profound complex disease study.

## Materials and Methods

### Human lincRNA data

We obtained 6,631 human lincRNAs with genomic coordinates from the lincRNA list of GENCODE project (version 16) [26], and removed lincRNAs without unique determinate chromosomal location. Finally, 5,835 lincRNAs were contained in SNP@lincTFBS.

### Identifying conserved TFBSs of human lincRNAs

We downloaded the locations and scores of conserved TFBSs from the UCSC genome browser [27]. These data were obtained by running the program tfloc (Transcription Factor binding site LOCater) on multiz46way alignments, restricting only to the July 2007 (mm9) mouse genome assembly, the November 2004 rat assembly (rn4), and the February 2009 human genome assembly (hg19). A binding site is considered to be conserved across the alignment if its score meets the threshold score for its binding matrix in all 3 species (human, mouse and rat). Transcription factor information was downloaded from the Transfac Factor database, and the score and threshold were computed with the Transfac Matrix Database (v7.0) created by Biobase [28]. Then, We defined 5 kb upstream to 1 kb downstream region of the transcription start site of each lincRNA as its promoter region refer to previous study [29]. We identified the conserved TFBSs of human lincRNAs in these regions; as a result, we identified 33,181 TFBSs in defined promoter regions of 3,839 human lincRNAs.

### Identifying TFBSs of lincRNA using genome-wide ChIP-Seq data

We downloaded 690 ChIP-Seq datasets for 169 human transcription factors in different cell lines and tissues from ENCODE project [25]. These peak datasets were computed by a peak calling method (PeakSeq), which identified enriched peaks through comparing each ChIP-Seq dataset to corresponding control experiment [30]. Then, we identified the peaks that were located in the promoter regions of human lincRNAs (5 kb upstream to 1 kb downstream region of the transcription start site for each lincRNA). In total, we identified 323,256 transcription factor peaks of different transcription factors in 4,831 lincRNA promoter regions.

### Identifying SNPs in the TFBSs of human lincRNA

We downloaded SNPs (common and rare variants) in public dbSNP database (build ver. 137) and identified 6,665 SNPs within 6,614 putative TFBSs of 2,423 human lincRNAs. In addition, with ChIPSeq dataset, we identified 139,576 SNPs in 304,517 transcription factor peaks of 4,813 lincRNAs. Then, we downloaded the annotation information of minor allele frequencies and others from 1000 Genomes Project (release of July 2012) datasets across 11 populations [31], and performed comprehensive annotation for these SNPs in lincRNA TFBSs. For each SNP in a lincRNA TFBS, we also extracted the flanking sequence of 30 nt up-/down-stream of the SNP position from RefSeq reference genomic sequence.

### Collecting experimentally supported disease-associated SNPs in lincRNA TFBSs

We manually collected known disease-associated SNPs in lincRNAs TFBSs using PubMed to search the previous studies. We also annotated lincRNAs in SNP@lincTFBS that have been reported to be associated with diseases, and identified SNPs within their putative TFBSs. In addition, we integrated recently well-known disease-associated SNPs and disease lincRNAs into SNP@lincTFBS database.

### Database implementation

SNP@lincTFBS is an online query tool developed utilizing ECLIPSE platform as the frontend, and MySQL as the backend database. The web engine was implemented using JSP technology, Struts framework and the Java connection pool Proxool, and web server was built using Apache Tomcat.

## Results

### Overview of the SNP@lincTFBS Database

**Figure 1:**
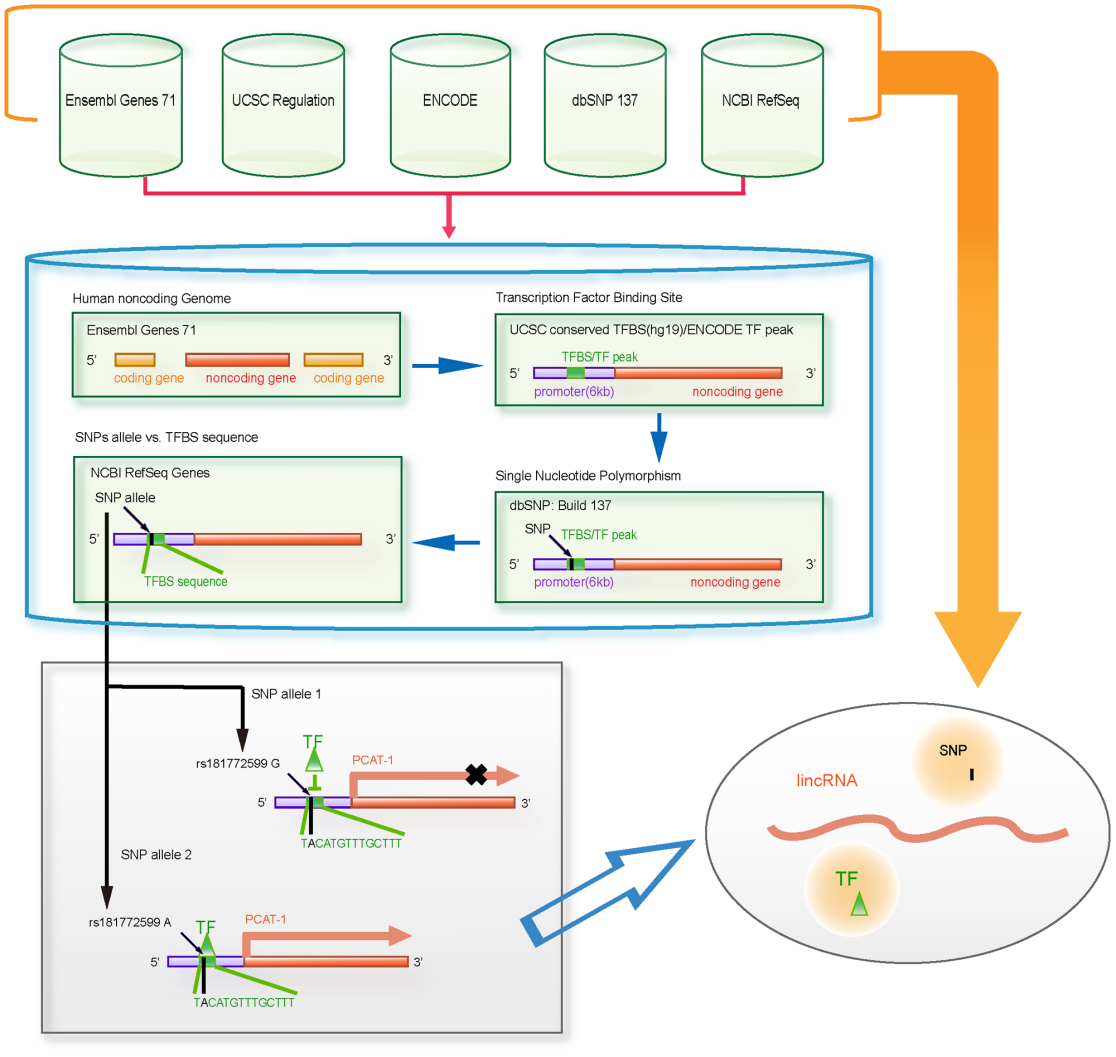
Architecture of SNP@lincTFBS.

We developed a novel integrated database named SNP@lincTFBS that allows users to perform SNP and TFBS searches in human lincRNAs. In this database, we: 1) obtained human lincRNAs, 2) identified conversed TFBSs and transcription factor peaks in defined promoter regions of human lincRNAs, 3) identified SNPs in the TFBS of lincRNA and collected experimentally supported disease-associated SNPs in lincRNA TFBSs, 4) integrated annotation information of SNP, TFBS and lincRNA. The architecture of identifying SNPs in lincRNA TFBSs is shown in Figure 1.

**Figure 2:**
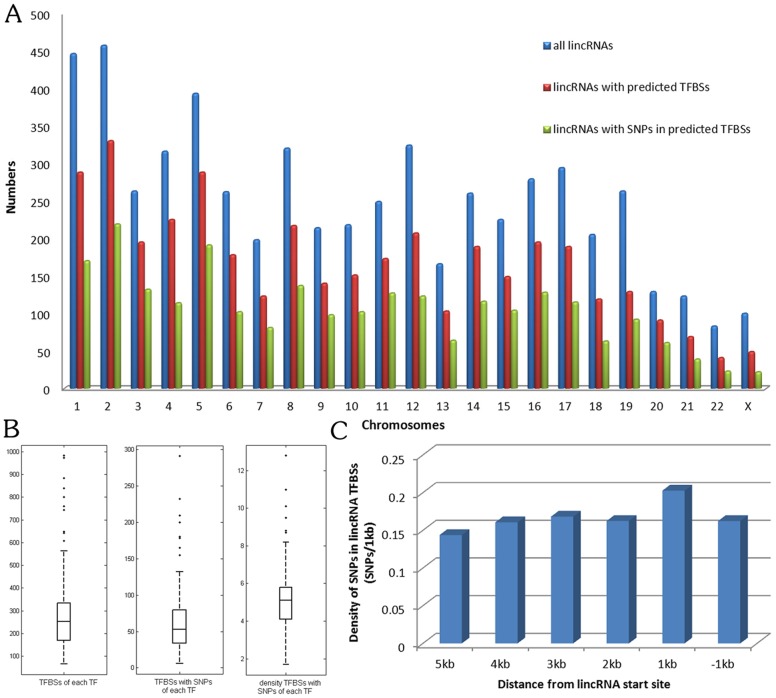
SNPs in human lincRNA TFBSs. (**A**) The number distribution of lincRNAs classified as chromosomes. Blue bars represent all lincRNAs. Red bars represent lincRNAs have TFBSs in their promoter regions. Green bars represent lincRNAs have SNPs in their TFBSs. (B) Statistics of lincRNA TFBSs with SNPs for each transcription factor. The quantity of lincRNA TFBSs for each transcription factor (left). The quantity of lincRNA TFBSs with SNPs for each transcription factor (middle). Density of lincRNA TFBSs with SNPs for each transcription factor (right). (C) Distribution of SNPs in lincRNA TFBSs with respect to distance to the lincRNAs. The x-axis displays the 1 kb window within 5 kb upstream to 1 kb downstream region of the start site of lincRNA and the y-axis displays the fraction of SNPs in lincRNA TFBSs located within this window.

Currently, SNP@lincTFBS contains 8,290 entries of annotated SNP-TFBS-lincRNA associations, including 3,839 lincRNAs, 33,181 conserved TFBSs, 6,665 SNPs and 165 transcription factors. In addition, 19,878,236 entries of SNP-peak-lincRNA associations were stored in SNP@lincTFBS, including 4,831 lincRNAs, 323,256 transcription factor peaks, 139,576 SNPs and 169 transcription factors. We identified a large number of conserved TFBSs in the promoter regions of human lincRNAs and found that the distribution of SNPs in these lincRNA TFBSs was extensive (Figure 2A). Previous studies have shown that each transcription factor can bind to several TFBSs in the promoter regions of protein-coding genes, thereby controlling the transcription of genetic information from DNA to messenger RNA. We also found a similar phenomenon in human lincRNA and a transcription factor could bind to many conserved lincRNA TFBSs (∼247 lincRNA), whereas ∼20 TFBSs that have been identified SNPs within them, and every 5.3 TFBSs had a SNP for each transcription factor (Figure 2B). In addition, we observed that high frequencies of SNPs within lincRNA TFBSs to be located around lincRNA start site (Figure 2C), suggesting that these SNPs within lincRNA TFBSs might greatly affect the expression of lincRNAs.

### Web interface

**Figure 3:**
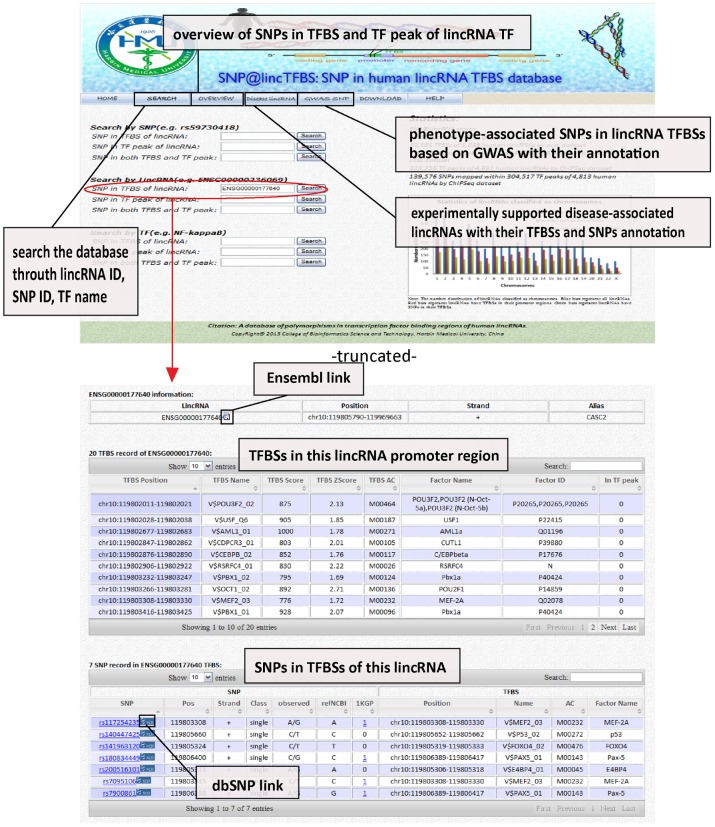
The homepage and an example of SNP@lincTFBS database. Screenshot of the main search page and corresponding result page, search as lincRNA ENSG00000177640.

The SNP@lincTFBS database website includes seven modules: home, search, overview, disease lincRNA, GWAS SNP, download and help (available at http://bioinfo.hrbmu.edu.cn/SNP_lincTFBS). HOME page provides a brief description of the SNP@lincTFBS database, users can browse the high-resolution flowchart of this work to get the main idea of this database. SEARCH page provides a quick search by query of three kinds of entries: 1) a lincRNA name (Ensembl ID), 2) an SNP identifier (rs number from dbSNP), and 3) a transcription factor name. Statistic of dataset contained in the database is introduced. Search result shows lincRNA summary information and all identified TFBSs and TF peaks in promoter region of this lincRNA. SNPs in these TFBSs and TF peaks are listed below (Figure 3). OVERVIEW page provides a general overview of transcription factors stored in SNP@lincTFBS. Disease lincRNA page shows existing experimentally supported disease-associated lincRNAs with their annotations and internal links for their TFBSs and SNPs mapped within them. GWAS SNP page shows disease-associated SNPs from GWAS researches that can be mapped to the lincRNAs TFBSs, whole annotations about lincRNA and TFBS are also available by internal link. PubMed external link for relevant literature is provided. DOWNLOAD page allows users to download all data we provided at present, including TFBSs and TF peaks of lincRNA promoter regions and SNPs mapped within lincRNA TFBSs and TF peaks in the TXT format. HELP page provides detailed column label description of SNP@lincTFBS. Instruction and contact information are also obtained.

### Known disease SNPs in lincRNA TFBSs

The SNP@lincTFBS database was developed not only as a resource for identifying SNPs in putative TFBSs of human lincRNAs, but also as a direction for further confirmation of predicting novel disease-associated SNPs and lincRNAs. Previous studies have found that lincRNAs may tend to associated with the same diseases with the disease-associated SNPs within their TFBSs by affecting the expression of lincRNAs [21]. We found 22 known disease-associated SNPs in lincRNAs TFBSs using PubMed to search the previous studies (Table 1). For example, we found two SNPs, rs2001844 and rs6982502 in two predicted TFBSs of a lincRNA *ENSG00000253111*. These two SNPs were identified to be associated with the variation in the magnitude of statin-mediated reduction in total and LDL-cholesterol based on a genome-wide association study [32], thus this lincRNA might have a relationship with cholesterol-associated diseases. Further experimental validation of the role of these disease-associated SNPs in lincRNA TFBSs might provide new insights into mechanisms underlying human diseases.

**Table 1:** Disease-associated SNPs in lincRNA TFBSs.

Disease or phenotype	lincRNA	SNP	PubMed ID
Multiple complex diseases	ENSG00000204092	rs16868911	17554300
Celiac disease	ENSG00000224099	rs1542865	17558408
Multiple complex diseases	ENSG00000226029	rs11543230	17554300
Suicide attempts in bipolar disorder	ENSG00000227336	rs4886217	21423239
Obesity (extreme)	ENSG00000228153	rs17413714	21935397
Multiple complex diseases	ENSG00000228590	rs9309325	17554300
Suicide attempts in bipolar disorder	ENSG00000228909	rs7587562	21423239
Multiple continuous traits in DGI samples	ENSG00000230812	rs2716133	17463246
Type II Diabetes Mellitus	ENSG00000233081	rs12683158	17463248
Multiple continuous traits in DGI samples	ENSG00000241884	rs9856163	17463246
Progressive supranuclear palsy	ENSG00000251009	rs1545606	21685912
Response to statin therapy	ENSG00000253111	rs2001844	20339536
Coronary heart disease	ENSG00000253111	rs6982502	22319020
Response to statin therapy	ENSG00000253111	rs6982502	20339536
Urinary metabolites	ENSG00000253184	rs822249	21572414
Urinary metabolites	ENSG00000253248	rs1031282	21572414
Alzheimer's disease	ENSG00000253583	rs6472116	22005930
Type II Diabetes Mellitus	ENSG00000254822	rs12793795	17463248
Major depressive disorder (broad)	ENSG00000259284	rs8028149	20038947
Childhood asthma	ENSG00000264968	rs4065275	17611496
Multiple complex diseases	ENSG00000267416	rs12951337	17554300
Serum urate	ENSG00000269290	rs493573	21768215

We also found several lincRNAs in SNP@lincTFBS that have been reported to be associated with human diseases, and these lincRNAs had SNPs within their putative TFBSs. For example, we found human lincRNAs NAG7, MEG3, PCAT1, CASC2 and LINC00032, which were involved in nasopharyngeal carcinoma [33], glioma and bladder cancer [34], [35], prostate cancer [36], endometrial cancer [37] and melanoma [38]. We identified several SNPs in the TFBSs of these disease-associated lincRNAs. These SNPs might be potential risk SNPs for diverse diseases by regulating the expression of disease-associated lincRNAs. For example, the research on *NAG7* gene involved in human nasopharyngeal carcinoma (NPC) susceptibility can be traced to more than a decade, and previous studies have found that *NAG7* played a key role by means of both expression and interaction, it could inhibit proliferation and induce apoptosis in NPC cell but also stimulate NPC cell invasion [22], [33], [39]. Soon after, *NAG7* gene was provided as a long intergenic non-protein coding RNA 312 (*LINC00312*) in HGNC (HUGO Gene Nomenclature Committee) [40]. Recently, an investigation aiming to assess the possible correlations of *LINC00312* expression with NPC progression based on microarray technology has indicated that *LINC00312* was significantly down-regulated in NPC tissues and it could represent a potential biomarker for metastasis, progression and prognosis in NPC [41]. In the SNP@lincTFBS database, we found a SNP (rs112175570) located within the TFBS for the transcription factor NF-κB and RelA in the promoter of *LINC00312* gene (Ensembl ID: ENSG00000237697), and rs112175570 might be a potential risk SNP for nasopharyngeal carcinoma by regulating the expression of *LINC00312*.

Besides cancer, we also found several neurological or psychiatric disorder associated SNP in human lincRNA TFBSs. For example, we found three SNPs (rs141600967, rs111946796, rs147394431) in the TFBSs of a lincRNA, ENSG00000214548 (also known as MEG3), ENSG00000214548 has been demonstrated to be associated with multiple human diseases, including glioma and neuroblastoma [42], [43]. We found three SNPs (rs2973034, rs2973034, rs78670708) in the TFBSs of a lincRNA, ENSG00000248587 (also known as GDNF-AS1), ENSG00000248587 has been demonstrated to be associated with Alzheimer disease [44]. In addition, we found a Alzheimer's disease risk SNPs (rs6472116, p = 9.59×10^−5^) in a lincRNA TFBS (ENSG00000253583) [45]. Therefore, further experimental verification of this SNP might provide novel insights and lead to new treatments. Taking advantage of our database, it is possible to further investigate the mechanism of lincRNA involved in human diseases.

## Discussion

Accumulating studies of dysregulated lincRNA expression in diverse cancers have suggested that lincRNAs might act as potential tumor suppressor genes and novel prospective therapeutic targets in cancer treatments. SNP@lincTFBS is designed to serve as a practical resource of SNPs in the TFBSs that dysregulate the expression of human lincRNAs. The database provides available genomic informations and annotations of SNPs in the TFBSs in putative promoter regions of human lincRNAs, and also a web-based interface allowed easy access to query and download flexibly. Most human lincRNAs have TFBSs in their promoter regions and the distribution of SNPs in these TFBSs of lincRNAs is widespread.

Previous studies have demonstrated that the genetic variants in the TFBSs of human lincRNA regulatory regions may change lincRNA expression, and thereby affecting the susceptibility to human diseases [21]. Thus we developed the SNP@lincTFBS database, which is devoted to the exploration and annotation of SNPs in potential TFBSs of human lincRNAs. One of the distinctive features of SNP@lincTFBS is that all SNPs that can be mapped to human lincRNA TFBSs are identified and annotated. The other databases that are related to transcriptional regulation for lncRNAs, such ChIPBase [24], only collect TF-lncRNA regulatory relationships that have been identified from ChIP-Seq data. In SNP@lincTFBS, we considered not only transcription factor of lincRNAs (like ChIPBase), but also the SNPs that affect the capability of binding to the lincRNA promoter regions of each transcription factor.

Our database has the potential to become an available resource for further studies of lincRNA function and complex disease. For example, we found several disease-associated SNPs and lincRNAs in SNP@lincTFBS, suggested the potential application of the SNP@lincTFBS in the field of disease-associated lincRNA variants. We found multiple SNPs in the TFBSs of cancer-associated lincRNAs, further experimental verification of these disease candidates might yield novel insights into disease pathophysiology. In addition, we also found multiple SNPs in the TFBSs of neurological or psychiatric disorder associated lincRNAs, this finding was consistent with previous studies, which revealed that lincRNAs played important roles in brain [5] and neuropsychiatric disorders [46]. Although the current number is limited, with the growth of interest in human lincRNAs and the availability of high-throughput technologies, the total number of disease-associated lincRNAs and SNPs will undoubtedly continue to grow, SNP@lincTFBS will become increasingly useful in future studies.

In the future, we envisage the database to be available as a semantically linked interoperable data resource. We hope that SNP@lincTFBS will be a useful tool for researchers in pertinent fields, and will benefit the functional study of human lincRNAs. With the increasing availability of genome-wide transcriptome identification and functional annotation of human lincRNAs in the public domain, we would enrich the database with this information. We will update the disease-associated lincRNAs with their annotations and disease-associated SNPs mapped to the TFBSs of lincRNAs every 4 months. SNP@lincTFBS may act as an advance resource that can provide great convenience for the research on identification of disease-associated lincRNAs or risk SNPs and the discovery of responsibility for discrepant expression abundance of lincRNAs.
